# Malaria case detection using rapid diagnostic test at the community level in Ghana: consumer perception and practitioners’ experiences

**DOI:** 10.1186/s12936-016-1086-z

**Published:** 2016-01-22

**Authors:** Daniel A. Danquah, Kwame O. Buabeng, Kwaku P. Asante, Emmanuel Mahama, Constance Bart-Plange, Ellis Owusu-Dabo

**Affiliations:** Department of Clinical and Social Pharmacy, Faculty of Pharmacy and Pharmaceutical Sciences, College of Health Sciences, Kwame Nkrumah University of Science and Technology, Kumasi, Ghana; Education and Training Department, Pharmacy Council, Accra, Ghana; Kintampo Health Research Centre, Ghana Health Service, Kintampo, B/A Ghana; National Malaria Control Programme, Ghana Health Service, Accra, Ghana; Kumasi Centre for Collaborative Research, College of Health Sciences, Kwame Nkrumah University of Science and Technology, Kumasi, Ghana

**Keywords:** Malaria, Perception, Experiences, Rapid diagnostic test, Medicine outlets

## Abstract

**Background:**

Ghana has scaled-up malaria control strategies over the past decade. Much as malaria morbidity and mortality seem to have declined with these efforts, there appears to be increased consumption of artemisinin-based combination therapy (ACT). This study explored the perception and experiences of community members and medicines outlet practitioners on malaria case detection using rapid diagnostic test (RDTs) to guide malaria therapy.

**Methods:**

This was a cross-sectional study using both quantitative and qualitative approaches for data. In-depth interviews with structured questionnaires were conducted among 197 practitioners randomly selected from community pharmacies and over-the-counter medicine sellers shops within two metropolis (Kumasi and Obuasi) in the Ashanti Region of Ghana. Two focus group discussions were also held in the two communities among female adult caregivers.

**Results:**

Medicine outlet practitioners and community members often used raised body temperature of individuals as an index for malaria case detection. The raised body temperature was presumptively determined by touching the forehead with hands. Seventy percent of the practitioners’ perceived malaria RDTs are used in hospitals and clinics but not in retail medicines outlets. Many of the practitioners and community members agreed to the need for using RDT for malaria case detection at medicine outlets. However, about 30 % of the practitioners (n = 59) and some community members (n = 6) held the view that RDT negative results does not mean no malaria illness and would use ACT.

**Conclusions:**

Though malaria RDT use in medicines outlets was largely uncommon, both community members and medicine outlet practitioners welcomed its use. Public education is however needed to improve malaria case detection using RDTs at the community level, to inform appropriate use of ACT.

## Background

Prompt recognition and effective treatment of malaria is a critical element of the strategies for malaria control in endemic countries [1]. The scaling up of malaria RDTs at all levels of health care is essential to ensure early case detection and appropriate management of malaria [2]. Since 1998, Ghana has committed itself to the Roll Back Malaria Initiative of the World Health Organization with a goal to reduce the global malaria burden by 75 % by 2015 [2]. This initiative also targets universal access to malaria diagnostic testing in public and private sector facilities as well as at the community level [2]. However, in most malaria endemic countries, reliable malaria case detection is limited and misdiagnosis commonly occurs exacerbating morbidity and promoting poor perception of malaria [3, 4].

Some misconceptions about the causes of malaria such as eating fatty meals, standing in the sun and the lack of proper hygiene reported in studies conducted in 1992 [5] was again reported in similar studies under similar socio-cultural context in Ghana in 2010 [6]. These poor perceptions of the community about malaria may have contributed to the challenges in malaria control in Ghana and other malaria endemic countries.

Malaria case detection using RDTs is successfully being implemented in hospitals and clinics in Ghana over the past 6 years. However, there are common perceptions and beliefs about the accuracy of these tests and their impact on patient care in places where such tests are provided. There are reported uses of anti-malarial medicines in non-malaria febrile illnesses even when RDT was conducted [7–11]. Understanding the experiences and perceptions of the community on malaria case detection will help target malaria RDT use interventions at the community level.

Even though there has been a global call for parasitological confirmation, either by malaria microscopy or RDT for patients of all ages with suspected malaria at all levels of healthcare [12], medicine outlets are yet to be fully introduced to test-based management of malaria in Ghana. Though published literature on the perception of malaria case management in medicine outlets is poor [13–15], effectively exploring the perceptions of the community and experiences of practitioners and designing interventions that take into consideration these perceptions and experiences may improve the appreciation of RDT use protocols and guidelines at the community level [16]. It is therefore necessary to continuously explore people’s perception and experiences with malaria case detection using RDTs since among other factors, the acceptance of any healthcare intervention depends on people’s perception of that innovation [17]. In this study, the perceptions and experiences of medicine outlet practitioners on malaria case detection and that of people in the community were explored, and how that informed the use of ACT for malaria therapy.

## Methods

### Study area

The study was conducted in two urban districts of Ashanti region namely Kumasi Metropolitan Area (KMA) and Obuasi Municipal Area (OMA). These two districts have similar socio-cultural background and relatively higher economic activity than the other districts in Ashanti region [18]. Both settings have had several malaria prevention and control programmes instituted including, intermittent preventive treatment in pregnancy, the hang-up campaigns during which Long-lasting insecticide-treated nets (LLINs) were distributed to all households in the Ashanti Region and the Indoor Residual Spraying project by AngloGold Ashanti in collaboration with the National Malaria Control Programme and the Obuasi Municipal Assembly complementing malaria prevention and control strategies in Ghana since 2005 [19]. Though the study area have several hospitals and clinics, the community members largely seek healthcare from medicine outlets initially before visiting hospitals and clinics [20].

Medicine outlet refers to either a community pharmacy or an OTCMS shop and medicine outlet practitioners included pharmacists, pharmacy technicians, medicine counter assistants, OTCMS and OTCMS assistants. The OTCMS shops are auxiliary pharmaceutical facilities licensed for the supply by retail only, over-the-counter medicines by the Pharmacy Council. They constitute about 80 % of the total number of private medicines outlets and are found in almost every town or village whereas community pharmacies are mainly found in urban communities of Ghana [21]. At the time of this study, all pharmacists in Ghana had been trained in RDT use by the National Malaria Control Programme in collaboration with the Pharmacy Council.

### Study design

The study design was cross-sectional, using a mixed approach of both qualitative and quantitative methods. The qualitative study consisted of focus group discussions (FGDs) among community members to assess their perceptions on malaria case detection using RDTs. The quantitative component preceded the qualitative study and consisted of structured questionnaires administered to practitioners in the systematic randomly selected medicine outlets from a list of all medicines outlets obtained from the Pharmacy Council, the national legal entity that registers all medicine outlets in Ghana.

### Study procedures

#### Quantitative methods

Structured interviews were conducted among staff of 197 medicine outlets selected from a total of 1032 using a simple random selection. Ten trained interviewers who have a degree in pharmacy from the Kwame Nkrumah University of Science and Technology conducted the interviews. Only practitioners present at the time of visit and willing to participate were interviewed using structured questionnaire. Each interview was conducted in English at the premises of the medicine outlet and lasted about an hour.

#### Qualitative methods

FGDs were held in each of the districts at locations that ensured privacy and the comfort of participants. Participants were selected from households located within one kilometre radius to the medicine outlets. The study participants were aged between 25 and 56 years and were purposively chosen to include female care-givers or parents with children not less than 5 years and speak at least one common language. Two FGDs were conducted using the local dialect ‘Twi’ with eight participants in each discussion. The principal investigator facilitated the discussions while a trained interviewer took notes. The discussions were also recorded on audiotapes with the consent of participants.

### Sample size calculation

The sample size estimation for practitioner interview was based on the proportion of medicine outlets using RDTs in malaria case detection as per the national malaria case management guidelines. The experiences on RDT use in malaria case detection of staff of medicine outlets in Ghana is not widely documented. Assuming 50 % of OTCMS had had an experience with RDTs and a hypothesized proportion of 60 %, a sample size of 197 provides 80 % power to determine from the true population proportion of pharmacies and OTCMS shops which have experienced the use of RDTs to detect malaria cases at 95 % confidence level. In the qualitative study, two FGDs were purposively conducted and did not fulfil and power and sample size estimation approach being qualitative.

### Data entry, cleaning and analysis

The quantitative data collected in the field were checked for discrepancies and completeness. The questionnaires were logged for traceability, and then batched for double data entry using Microsoft Visual FoxPro 9.0, data management software. All data management processes were done at the Kintampo Health Research Centre, one of the three research centres under the Research and Development Division of the Ghana Health Service. Data analysis was done using StataCorp Stata 12.0, TX USA.

All variables that were categorical in nature were summarized as proportions while continuous variables expressed as means together with their respective standard deviations.

The qualitative data were tape-recorded and manually transcribed verbatim by three female pharmacy graduates who were not part of the initial data collection process. Participants’ responses to the discussion guide were grouped and categorized using NVIVO version 8 Software. Themes that were related to the study objectives were identified and the corresponding quotes that best described the themes were recorded to support the study findings. Demographic data on respondents such as ages, sex and educational background were summarized as percentages.

### Ethical approval

Ethical approval was obtained from the Committee of Health Research, Publication and Ethics of the College of Health Sciences, KNUST (CHRPE/AP/205/13). Informed consent was also obtained from all the participants before recruitment into the study. Only individuals who consented were recruited for the study and provided with all the necessary information regarding the study aims, objectives, risk and benefits associated with the study. To ensure confidentiality, all data collected were stored in locked cabinets at the Kintampo Research Centre.

## Results

### Socio-demographic characteristics of respondents

Sixty six percent of the practitioners (n = 130) were from OTCMS shops while 34 % were from community pharmacies (n = 67). Sixty two percent were assistants either working in pharmacies (n = 43) or in OTCMS shops (n = 79). Forty seven percent of the service providers in the outlets (n = 92) were aged 18–30 years whilst 22 % of them (n = 44) were 51 years and above. Highest educational level attained by 57 % of the practitioners was secondary and 33 % had tertiary education. Forty six percent (n = 90) had worked as practitioners for not more than 5 years as shown in Table 1.

**Table 1:** Demographic characteristics of practitioners interviewed

Characteristics (N = 197)	n	%
Facility type		
Community pharmacies	67	34.0
OTCMS shops	130	66.0
Practitioner type		
Pharmacists	11	5.6
Pharmacy technicians	13	6.6
Medicine counter assistants	43	21.8
OTCMS	51	25.9
OTCMS assistants	79	40.1
Highest educational level		
Tertiary	64	32.5
Secondary	113	57.4
Basic (up to JHS)	18	10.1
Age (years)		
18–30	92	46.7
31–40	31	15.7
41–50	30	15.2
51 and above	44	22.3
Years worked as a service provider		
1–5	80	42.0
6–10	41	21.0
11–20	41	21.0
21 and above	25	13.0

The FGD participants were aged between 24 and 56 years. Six out of the 16 participants had basic education, six completed secondary education and three had never been to school. More than half of the respondents were either petty traders or employees of both public and private institutions while the others were unemployed (Table 2).

**Table 2:** Demographic details of FGD participants

Participant ID	Age	Marital status	Highest level of education	Occupation	Number of children
OMA					
BG145	41	Married	Secondary	Unemployed	3
BG171	35	Married	Secondary	Trader	2
MBK41	52	Married	Uneducated	Trader	4
MBK45	25	Married	Basic	Hairdresser	1
BG174	46	Married	Basic	Unemployed	3
KNT44	24	Married	Basic	Unemployed	1
KNT104	43	Married	Basic	Trader	3
KNT144	30	Married	Secondary	Teacher	1
KMA					
FNT100	47	Married	Basic	Unemployed	3
AB17	50	Married	Secondary	Trader	4
AB141	39	Married	Uneducated	Trader	2
AT45	28	Married	Basic	Hairdresser	2
AT134	56	Married	Uneducated	Unemployed	3
FNT20	27	Married	Secondary	Unemployed	1
BT14	44	Married	Basic	Trader	4
BT142	38	Married	Secondary	Civil servant	2

### Respondents perception and experiences on malaria case detection

Majority of the practitioners (132/195) knew about malaria case detection using RDTs however, their experiences were in hospitals and clinics, but not in medicine outlets (Table 3).

**Table 3:** Awareness of malaria RDT among medicine outlet practitioners

Characteristics (N = 195)	n	%
Awareness of RDTs		
Community pharmacies	62	94.0
OTCMS shops	70	54.0

When the FGD participants were asked how they detect malaria cases, many associated malaria with raised body temperature. Other signs relied upon were headache, sweating, itching, insomnia and bitterness of the mouth.

When the body feels very hot then you know you have malaria (FGD Obuasi, BG174)

When your mouth tastes bitter and you feel uneasy, you will not suspect any other disease but malaria (FGD Kumasi, BT142).

Personally when I’m about to get malaria, I get itchy all over my body especially after bathing (FGD Kumasi, AT134).

Most practitioners indicated that they detect malaria cases presumptively relying solely on the signs and symptoms such as fever, headache that the client presents. When the perception of the practitioners was sought on the prevalence of fever and malaria among visiting clients, the practitioners believed that 58 % of clients visiting medicine outlets had fever whilst 57.5 % had malaria (Table 4). There is also a strong positive correlation (R = 0.84) between malaria and fever (Table 4).

**Table 4:** Relationship between practitioners’ perception of malaria and fever

	Facility type		
	Pharmacy (N = 67)	OTCMS shop (N = 128)	P
	Mean (95 % CI)	Mean (95 % CI)	
Malaria	7.54 (4.71–10.36)	11.20 (7.44–14.95)	0.1942
Fever	10.21 (5.45–14.97)	11.44 (7.45–15.42)	0.708

Perception	n (%)	n (%)	P
Malaria prevalence	59	56	0.26
Fever prevalence	60	56	0.25

Correlation coefficient (R) = 0.8407; P < 0.001

### Health seeking behavior of community members

Community members indicated they usually treat themselves at home when they suspect they have malaria. Others proceed to the nearest pharmacy or OTCMS shop for anti-malaria therapy and when no improvement in their health condition is observed then they decide to go to a clinic or hospital.

I first go to the drug store. If I do not get better a day or two after visiting the drug store, I proceed to the hospital (FGD Obuasi, KNT44).

### Perception and experiences on malaria RDT use in medicine outlets

**Fig. 1:**
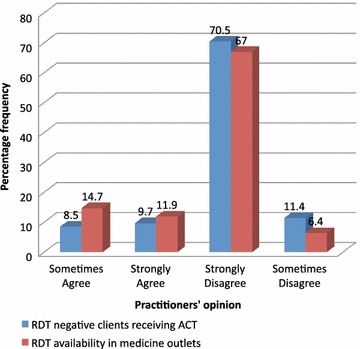
Practitioners’ opinion on access and adherence to RDT results

Seventy percent (137/195) of the practitioners were of the view that malaria RDTs are not available in medicine outlets (Fig. 1). Community members at both FGDs unanimously confirmed the absence of RDT services in medicine outlets as below:

None of us had experienced the use of malaria RDT in any medicine outlet… (All participants at both FGDs in Obuasi and Kumasi).

They further shared their experiences when they sent their family members to the hospital. They appreciated that a febrile illness is not always caused by malaria:

I sent my mother to the hospital last week and they did the RDT test for her. I also realized that they did it for every patient who came there. The test showed negative for the malaria parasite…. (FGD Obuasi, KNT144).

My fourteen years old son had frequent episodes of fever, he was tested and it showed negative for malaria but he later tested positive for typhoid fever (FGD Kumasi, AB17).

When medicine outlet practitioners were asked about what they will do to clients testing negative to the RDT, about 18 % (n = 36) of the practitioners were ready to administer anti-malaria treatment despite the RDT negative results (Refer Fig. 1). Similarly, the FGD participants were asked about what they will do if they tested negative. Below are quotes from the FGD participants:

If there is no parasite in the blood I would not buy any anti-malaria drug but rather I would purchase a drug for whatever disease is presenting from the test (FGD Obuasi, BG145).

If the test is negative I would still purchase and use the anti-malaria drug for prevention of the disease (FGD Kumasi, AT45).

When the community members and the practitioners were asked whether they will accept or render malaria RDT services in medicines outlets, both the community members and the practitioners welcomed its use in medicine outlets. However, the FGD participants expressed concern about the need to train all personnel working in the medicines outlets before its introduction. The following were quotes from the participants:

I will be happy if my temperature is measured and the malaria test done when I go to the drug stores, in that way I will feel well taken care of, just like when I go to the hospital (FGD Obuasi, MBK41).

We spend so much time at the hospital because we have to join long queues, it would be best if the drug stores have the testing kits so that we can effectively test and treat malaria (FGD Obuasi, BG171).

We want to be assured that each patient is pricked with a different needle during the test as it is done in the hospital (FGD Kumasi, FNT100).

### Respondents’ perception and experiences on malaria

Over 70 % of the FGD participants indicated that malaria is a disease that results from the bite of mosquitoes. However, some had misconceptions about the mode of malaria transmission. Some were of the view that malaria could be transmitted through contact with faeces, or excessive intake of oily or fatty meals and lack of proper personal hygiene. Similarly, the practitioners also attributed the cause of malaria to bites from infected female Anopheles mosquitoes.

## Discussion

Malaria case detection and treatment in Ghana like many other malaria endemic countries has been largely presumptive over the years especially in medicine outlets [4, 22]. At the community level, malaria case is usually presumed to be fever with the other symptoms like headache. In this study, six out of every ten febrile clients visiting medicine outlets were perceived to have malaria (Table 4). This perception probably assumes that all fevers reported at medicine outlets were appropriately detected and that the fevers are due to the malaria infection. Though there is a strong positive correlation between fever and malaria as indicated in Table 4, a fever case in the medicine outlets were mainly reported but not objectively measured. It is therefore important for medicine outlets to actually measure temperatures using thermometers.

The perception of community members about malaria may affect their ability to promptly and correctly identify a suspected malaria case. Even when a suspected malaria case is identified, the perception and experiences of the community and the practitioners may affect their appreciation of the RDT use protocols and guidelines. Eighteen percent of the practitioners (Fig. 1) and about 30 % of the community members indicated they would use anti-malarial medicines even when their RDT result is negative. This observation is similar to results of other studies conducted in Burkina Faso, Tanzania, Kenya and Ghana [7–11].

Though a good number of the practitioners were aware of malaria RDT use, this experience was seen in hospitals and clinics but not in community-based medicine outlets (Table 3). The low level of experience about malaria case detection using RDTs in medicine outlets may influence its use to inform malaria therapy, as medicines outlets are usually the first port of call for health seeking when people are unwell initially before visiting hospitals or clinics. Both the medicine outlets practitioners and the community members interviewed were rather aware of malaria testing in hospitals probably because the use of RDTs at the community level was uncommon. Both the community and the practitioners largely recognized the importance of the RDT results in malaria case detection and treatment. The participants accepted in principle the introduction of malaria RDT use in medicines outlets provided, the practitioners would be properly trained and supervised as reported in similar studies conducted in Ghana and Uganda [23, 24]. This seeming lack of trust in medicine outlets practitioners has been reported in similar studies in Nigeria and Tanzania [4, 10, 25, 26]. There is the need to train medicine outlet practitioners and sensitize community members to ensure public confidence in them.

Though the number of the FGDs held was not large enough to make definitive generalization that go beyond the study communities, the emerging themes were the same in both discussions and similar to that of other studies [4–6]. For instance, the misconception about malaria transmission, the emphasis on training and supervision of practitioners before introduction of RDT in medicine outlets and the level of adherence to the test results were similarly raised in both discussions. The study outcome therefore provides insights among others into some of the community perceptions and experiences on malaria case detection and control. For instance, it is easier for a client without fever to accept his or her RDT negative results than a client with fever. Similarly, the LLIN acquisition and use campaign may have lesser impact in communities who perceive malaria to be caused by excessive eating of fatty meals or of any other reason.

## Conclusions

The experience of community members and medicine outlet practitioners on the use of RDTs is low, and where they exist, mainly limited to clinics and hospitals. However, both the community members and medicine outlet practitioners welcome the use of RDTs in detecting malaria cases at the community level in Ghana. The inappropriate association of fever to malaria case detection may have serious health implications in particular on the use of ACT, especially when the fever is presumptively detected and treated. There is, therefore, the need to continue efforts aimed at avoiding all forms of presumptive malaria case detection and treatment in place of standard procedures in Ghana.

## Abbreviations

ACT: artemisinin-based combination therapy
RDTs: rapid diagnostic tests
KMA: Kumasi Metropolitan Area
LLINs: long-lasting insecticide-treated nets
OMA: Obuasi Municipal Area
OTCMS: over-the-counter medicine sellers
PC: Pharmacy Council
FGDs: focus group discussions
GNA: Ghana News Agency
